# Characterization of Biodegradable Polymers for Porous Structure: Further Steps toward Sustainable Plastics

**DOI:** 10.3390/polym16081147

**Published:** 2024-04-19

**Authors:** Guilherme M. R. Lima, Adrivit Mukherjee, Francesco Picchioni, Ranjita K. Bose

**Affiliations:** Product Technology Department, University of Groningen, 9747 AG Groningen, The Netherlands; g.de.macedo.rooweder.lima@rug.nl (G.M.R.L.); a.mukherjee@rug.nl (A.M.); f.picchioni@rug.nl (F.P.)

**Keywords:** sustainability, biodegradable, foam, bioplastics, supercritical carbon dioxide

## Abstract

Plastic pollution poses a significant environmental challenge, necessitating the investigation of bioplastics with reduced end-of-life impact. This study systematically characterizes four promising bioplastics—polybutylene adipate terephthalate (PBAT), polybutylene succinate (PBS), poly(3-hydroxybutyrate-co-3-hydroxyvalerate) (PHBV), and polylactic acid (PLA). Through a comprehensive analysis of their chemical, thermal, and mechanical properties, we elucidate their structural intricacies, processing behaviors, and potential morphologies. Employing an environmentally friendly process utilizing supercritical carbon dioxide, we successfully produced porous materials with microcellular structures. PBAT, PBS, and PLA exhibit closed-cell morphologies, while PHBV presents open cells, reflecting their distinct overall properties. Notably, PBAT foam demonstrated an average porous area of 1030.86 μm^2^, PBS showed an average porous area of 673 μm^2^, PHBV displayed open pores with an average area of 116.6 μm^2^, and PLA exhibited an average porous area of 620 μm^2^. Despite the intricacies involved in correlating morphology with material properties, the observed variations in pore area sizes align with the findings from chemical, thermal, and mechanical characterization. This alignment enhances our understanding of the morphological characteristics of each sample. Therefore, here, we report an advancement and comprehensive research in bioplastics, offering deeper insights into their properties and potential morphologies with an easy sustainable foaming process. The alignment of the process with sustainability principles, coupled with the unique features of each polymer, positions them as environmentally conscious and versatile materials for a range of applications.

## 1. Introduction

For many years, global pollution has been a primary concern impacting the planet [1]. Therefore, studying alternatives to substitute materials and processes is imperative, aiming for a more sustainable society. Plastics, in particular, have emerged as a significant environmental issue due to improper disposal, leading to contamination of land, air, and water [2,3,4]. In the modern era, envisioning a society without plastics seems nearly impossible [5]. Consequently, addressing pollution issues needs diverse solutions that have minimal impact on the co-evolution of materials and society. From a materials perspective, optimizing plastic recycling, extending the work life span of plastic products, using fewer materials for the same purpose, and incorporating biodegradability features have emerged as viable solutions. Among these, porous polymers made of biodegradable materials have garnered significant interest. However, adopting porous biodegradable polymers requires industries to modify their processes, study material properties, and carefully assess how biodegradability aligns with the intended purpose of the product.

Porous materials generally stand out as appealing products due to their minimal material requirements, large surface area, and relative ease of engineering to meet specific specifications [6,7]. These materials offer tunable features, including material density, energy insulation, mechanical properties, and light scattering [8,9]. Thus, beyond the matrix factor and formulation, porous materials allow for the introduction of novel features into the final product, such as biodegradability, biocompatibility, self-healing, shape memory, and electrical conductivity [10,11]. Porous polymers find applications across a broad spectrum of fields, including packaging, energy insulation, comfort, filters, and medical applications, such as tissue scaffolding or as carriers for the controlled release of pharmaceuticals [12,13]. In essence, the production of porous materials can be achieved through the use of chemical or physical blowing agents. Chemical agents, though easily controlled, often introduce new chemicals or raise toxicity concerns. Conversely, while more environmentally friendly, physical agents pose challenges in terms of control and process integration. A notable and environmentally sustainable method employing physical blowing agents involves utilizing CO_2_ [14,15]. The process unfolds through distinct steps: initially, CO_2_ is dissolved as a supercritical fluid in the molten polymer. Subsequent nucleation occurs over time as CO_2_ molecules disperse into the molten polymer. Finally, rapid pressure quenching reduces the force on dissolved gas molecules, allowing them to expand into bubbles, forming a porous structure within the molten polymer. This method is considered eco-friendly, avoiding the release of harmful chemicals and employing a green solvent that can be potentially recaptured and reused.

Biodegradable polymers, such as polybutylene adipate terephthalate (PBAT) and polybutylene succinate (PBS), stand out as potential groundbreakers for all industries with features matching non-biodegradable polymers. PBAT and PBS offer mechanical properties, thermal stability, and chemical resistance alongside their eco-friendly disposition [16,17,18]. Existing challenges, like pollution and fossil-based production methods, highlight the need for a comprehensive understanding of biopolymers’ properties and applications. PBAT is an amorphous aliphatic–aromatic copolyester and can be synthesized from the copolymerization of adipic acid, 1,4-butanediol, and dimethyl terephthalate [19,20]. Its notable mechanical properties, thermal stability, and biodegradability have produced some interest from the industry for possible PET replacement. PBS is a semi-crystalline aliphatic polymer derived from succinic acid and 1,4 butanediol and displays comparable overall properties with PBAT [21,22]. Some researchers [23,24,25] have shown that it is possible to produce engineered porous polymer materials with PBAT and PBS in their pure form or blends. Articles report tunable porous structures with fast biodegradability, low thermal conductivity, high compression strength, and low density. Some of the applications of PBAT porous materials involve medical purposes [26], aesthetic problems [27], and insulation [28]. The PBS porous structure is appointed for use as selective oil-adsorbing materials [29], packaging applications [30], and thermal insulation [31].

Plastic pollution leads to environmental imbalances and contributes to the depletion of finite fossil resources. It is essential to distinguish between biodegradability and being bio-based. While polymers from both capabilities exhibit great qualities for a more sustainable future, they are not mutually exclusive [32]. While pure PBAT and PBS, available in large quantities, are labeled as biodegradable and have the potential to be bio-based, they are currently manufactured using fossil-based chemicals. On the other hand, polylactic acid (PLA), a widely used polymer, is derived from lactic acid (LA) and represents a linear aliphatic thermoplastic polyester with various production options [33]. PLA can be synthesized through microbial fermentation, involving the purification of lactic acid and the preparation of its cyclic dimer. Another method includes the ring-opening polymerization of lactides, or polycondensation of lactic acid [34]. The versatility of porous PLA materials finds applications in diverse fields, such as medical purposes [35] and as a selective oil-adsorbing material [34]. In addition to PLA, polyhydroxyalkanoates (PHAs) represent a promising solution for addressing concerns related to petroleum reserves and biodegradability. PHAs are a class of polyesters produced by microorganisms, offering a non-toxic and biodegradable alternative. One notable member of the PHA family is poly(3-hydroxybutyrate-co-hydroxyvalerate) (PHBV), a biodegradable polyester characterized by low crystallinity, brittleness, and melting point compared to other PHAs. PHBV stands out for its biocompatibility, marine degradability, and mechanical properties similar to polyolefins [36]. PHBV is a copolymer, allowing for tunability based on the ratio of monomers. This characteristic enables the modification of diverse properties of the polymer, providing flexibility in tailoring its attributes for specific applications. Researchers have studied the porous structures of PHBV, reporting tunable morphology and closed and open cells, as well as high porosity in various studies [37,38]. These porous PHBV polymers hold promising applications, particularly in the medical field [39] and combating electromagnetic radiation pollution [40].

This study systematically characterizes four biodegradable polymers: PBAT, PBS, PHBV, and PLA. The comprehensive characterization covers chemical, thermal, and mechanical features to gain insights into their molecular structure, thermal behavior, and mechanical properties. Chemical characterization is crucial for verifying the molecular structure of the polymers and establishing correlations between their structures and subsequent properties. This step provides a foundational understanding of the intrinsic nature of each polymer. Thermal characterization is essential as it helps to understand the temperature transitions relevant to the foaming process and identifies key temperature points, such as degradation temperature. This knowledge is pivotal for optimizing the foaming conditions and ensuring the stability of the polymers during processing. The mechanical properties study is a critical component, offering insights into how each polymer is expected to behave during the foaming process and, by extension, in real-world applications. Understanding mechanical behavior is fundamental for predicting the structural integrity and performance of the porous materials produced. Subsequently, we transition to the production phase, making porous materials from each polymer sample. Utilizing similar processes with CO_2_ as a physical blowing agent, we seek to correlate the resulting morphologies with the previously characterized chemical, thermal, and mechanical properties. This integrative approach provides an integrated understanding of the polymers, shedding light on their behavior during foaming and the characteristics of the porous materials generated. Examining these biodegradable polymers aims to reveal their full potential and applications across various fields, including medical, filtering, comfort, protection, and insulation.

## 2. Materials and Methods

Poly(butylene adipate-co-terephthalate) (PBAT), trade name Ecoworld^®^ was obtained from Jinhui Zhaolong Co., Ltd., (Taiyuan, China). Poly(butylene succinate) (PBS), trade name PBE 003, was supplied by NaturePlast (Mondeville, France). Poly(3-hydroxybutyrate-co-3-hydroxyvalerate) (PHBV) was kindly provided by Paques Biomaterials (Balk, The Netherlands). Polylactic acid (PLA) (IngeoTM 8052D, D-isomer content: 4.7 mol%) was kindly provided by Foamplant B.V. (Groningen, The Netherlands). All materials were used as received without any additional modification or purification. Before processing, the PBAT, PBS, PHBV, and PLA material were dried under vacuum at 80 °C for a minimum of 5 h to remove any moisture residues.

### 2.1. Chemical Characterization

NMR spectra were recorded using a Varian VXR 400 MHz spectrometer (Palo Alto, CA, USA) at room temperature with samples dissolved in CDCl_3_ as solvent. Chemical shifts are reported in ppm and were calibrated to CDCl_3_, the main solvent. The collected spectra were analyzed using MestReNova (Mestrelab Research SL 12.0, Santiago de Compostela, Spain).

FTIR spectra were recorded with a Shimadzu IR-Tracer-100 (Kyoto, Japan) with a golden gate diamond attenuated total reflectance (ATR) sample unit in the range of 4000 cm^−1^ to 600 cm^−1^ at a resolution of 4 cm^−1^ averaged over 64 scans.

The molecular weights (*M_n_* and *M_w_*), as well as the polydispersity (PDI) of the samples, were measured relative to narrow-dispersity polystyrene standards in the range of 645 to 3 × 10^6^ g/mol using an SEC system equipped with a Viscotek GPCmax, GPC column oven (VE2585), and two PLgel MIXED-C (5 μm × 300 mm) analytical columns from Agilent Technologies (Santa Clara, CA, USA) with a separation range from 200 to 2 × $10^{6}$ g/mol thermostatically controlled to 35 °C in CHCl_3_ at a flow rate of 1.0 mL/min using a Schambeck RI2012 refractive index detector (Bad Honnef, Germany). For sample preparation, dry samples were dissolved in CHCl_3_, and, after they were completely dissolved, they were filtered through a polytetrafluoroethylene (PTFE) syringe filter (Minisart SRP 15, Sartorius stedim biotech, PTFE membrane filter; pore size, 0.2 μm; filter diameter, 15 mm) and analyzed using SEC.

### 2.2. Thermal Characterization

Thermal analysis was performed in a Perkin Elmer Pyris diamond 8000 (Shelton, CT, USA) differential scanning calorimeter instrument under a nitrogen flux of 50 mL/min to minimize oxidative degradation. Samples of approximately 5 mg were weighed in an aluminum pan and then sealed. The instrument was calibrated with a high-purity standard indium sample for melting temperature and heat of fusion. The samples were scanned in a temperature range according to each glass transition of the polymer and melting point by heating–cooling–heating cycles using a heating–cooling rate of 10 °C/min.

Thermogravimetric analysis was carried out in a Mettler-Toledo TGA analyzer under a nitrogen environment. A total of 10–20 mg of polymer sample was loaded in a crucible. During the TGA measurement, the temperature increased from 30 to 700 °C with a heating rate of 10 °C/min.

### 2.3. Mechanical Characterization

Each polymer sample was placed into a disc-shaped mold measuring 20 mm in diameter and 1 mm in thickness. Subsequently, the samples underwent compression and molding for a duration of 10 min under a force of 50 kN, with varying temperatures applied depending on the specific polymer being tested. The thermo-mechanical behavior of each sample was followed using a rotational rheometer (Discovery HR-2, TA Instruments, New Castle, DE, USA) in oscillation mode using 20 mm parallel plate geometry. An amplitude sweep was carried out first to ensure that the strain rate was within the linear viscoelastic (LVE) region for each polymer. Frequency sweep measurements were then conducted between frequencies of 0.1 and 500 rad/s with constant strain within the LVE region of each polymer. Temperature ramp tests were performed with strain under the LVE of each polymer and 1 rad/s.

### 2.4. Porous Polymer Processing and Analysis

Using the same protocol as for the disc-shaped mold in the mechanical characterization, the samples were placed into a different mold measuring 8 mm in diameter and 1 mm in thickness. These samples were specifically intended for foaming purposes. In order to achieve the porous material, batch foaming experiments were performed using CO_2_ as a blowing agent. The apparatus comprises a high-pressure pump and a high-pressure cell with temperature control. The procedure involved heating the reactor within the range of the polymer melting temperature, placing the samples in the cell, and flushing the cell with CO_2_ to remove any other gas in the chamber. Subsequently, the vessel was pressurized to 15 MPa using a high-pressure pump and left to soak for 30 min. After 30 min, the vessel was depressurized to atmospheric pressure in less than 3 s. The cross-sectional morphology of the cryo-fractured samples was imaged using scanning electron microscopy (SEM). The images were obtained from Nova NanoSEM 650 (Hillsboro, OR, USA) at a working distance of 5 mm and using an acceleration voltage of 10 kV. Before imaging, all samples were sputter-coated with 10 nm gold to avoid charging effects. The pore diameter distribution was subsequently obtained by analyzing the SEM images using Fiji software 2.9, where over 200 pore areas were individually measured for each sample [41].

## 3. Results

### 3.1. Chemical Characterization

**Figure 1:**
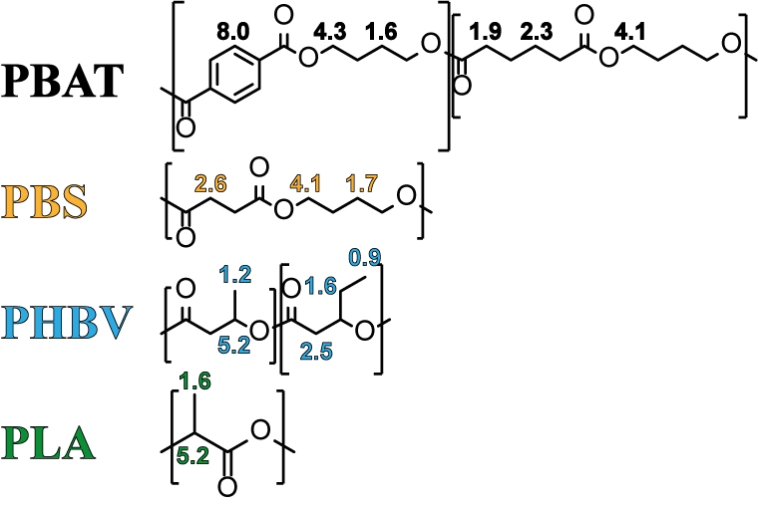
Chemical structure of PBAT, PBS, PHBV, and PLA with their respective protons assigned with the number corresponding to the NMR proton resonance signals in ppm.

**Figure 2:**
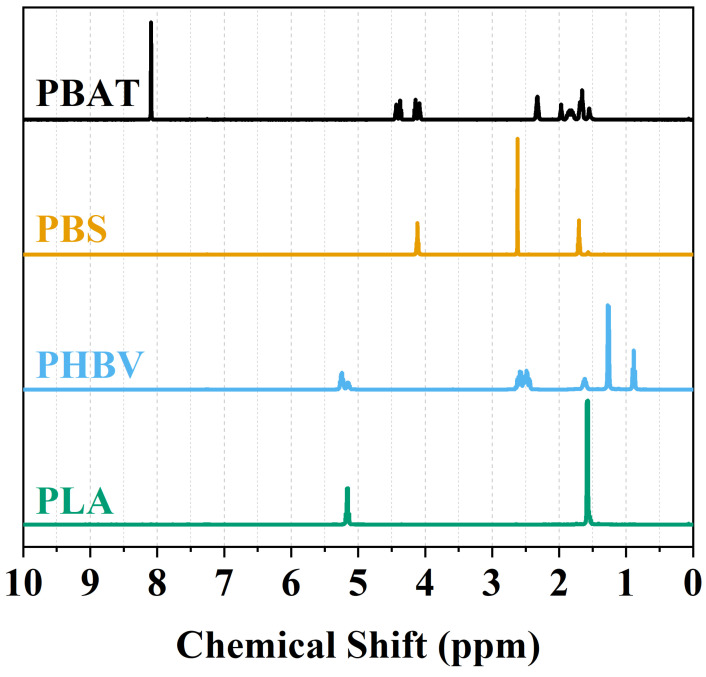
^1^H-NMR spectra of PBAT, PBS, PHBV, and PLA.

It is well known that polymer properties are intricately linked to factors such as monomer chemical structure, stereochemistry, chain length, and the distribution of the chain structure [42,43,44]. In this study, we employed the ^1^H-NMR technique to elucidate the chemical structure of each polymer. This method enabled the determination of the composition and molecular structure of each polymer sample. By obtaining NMR spectra and comparing them with the existing literature, we validated the pristine composition of PBAT, PBS, PHBV, and PLA polymers. Figure 1 shows the chemical structure of each biodegradable polymer with their respective NMR proton peaks in ppm. As shown in Figure 2, the ^1^H-NMR spectra of all samples are confirmed by the chemical structure in Figure 1 of each polymer by the presence of characteristic protons. The signal of aromatic protons of PBAT appears at 8.0 ppm, indicating the phenylene structure. Some signals of the CH_2_ are located at 4.3, 4.1, 2.3, 1.9, and 1.6 ppm as seen as a match with the chemical structure in Figure 1 and by previous studies [45,46]. PBS CH_2_ proton signals appear at 4.1, 2.6, and 1.7 ppm as indicated and confirmed in the literature [47,48]. Both PHBV CH signals are shown in multiple peaks at 5.2 ppm, where CH_2_ signals are also multiplets at 2.5, and 1.6 ppm, where the latter corresponds to the CH_2_ within the ethyl group. The CH_3_ has signals at 1.2 and 0.9 ppm, matching the literature [49,50]. The two proton signals of PLA appear at 5.2 ppm, indicating the CH signals, and at 1.6 ppm, indicating the CH_2_ matching the chemical structure in Figure 1 and other studies [51].

**Figure 3:**
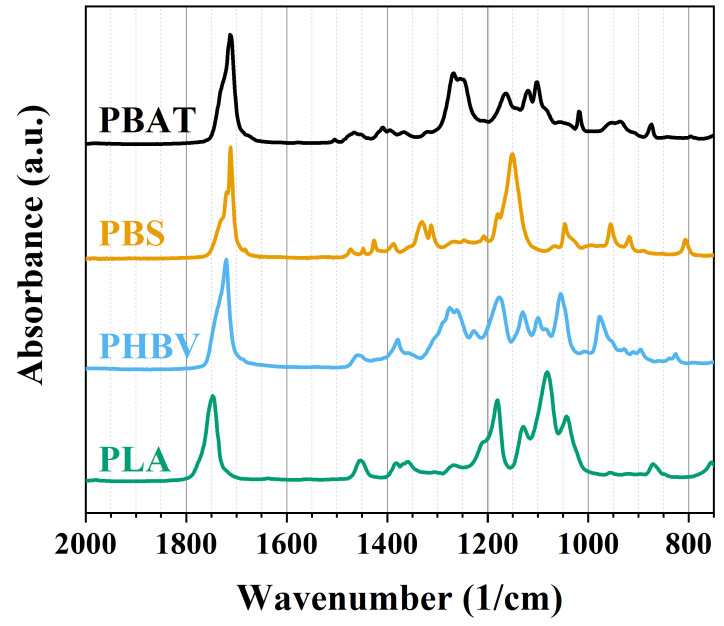
FTIR spectra of PBAT, PBS, PHBV, and PLA.

The FTIR shown in Figure 3 represents the structure of matter at the molecular scale measured from pristine PBAT, PBS, PHBV, and PLA. Compared with the literature, it detects and proves chemical composition and bonding arrangement. Since all four polymers studied in this article contain carbonyl groups CO, all four spectra display peaks at around 1720 cm^−1^. Specifically, for PBAT, it is appointed bands at 1504 cm^−1^, representing the skeleton vibration of the benzene ring, 1260 cm^−1^, representing CO in the ester linkage, and 1017 cm^−1^, referred to as the phenylene group. The band at 726 cm^−1^ represents the bending vibration absorption of the CH-plane of the benzene ring [16,17,52,53]. PBS main characteristic peaks were found at 1330 cm^−1^ for stretching vibration CH_2_, 1152 cm^−1^ for the stretching of COC, and band 1041 cm^−1^ for CO stretching [18,21,54]. PHBV spectra other than the carbonyl group show peaks at 1379 cm^−1^ correlated to stretching vibration CH_2_, 1152 cm^−1^ for the stretching of COC, and at 1041 cm^−1^ for CO stretching [36,55,56]. The chemical structure of PLA reveals distinct bands at 1187 cm^−1^, which signify the characteristic COC stretching in the ester groups. The peaks in PLA at 1360 cm^−1^, 1185 cm^−1^, and 1090 cm^−1^ are assigned to the bending vibration of CH, bending vibration of CH_3_, and stretching vibration of CCO, respectively [57,58].

Table 1 displays the molecular weights of all polymers determined through the GPC test. The polydispersity index (PDI) values for PBAT, PBS, PHBV, and PLA are 2.5, 2.3, 2.1, and 1.8, respectively. PBAT and PBS, synthesized via polymer condensation, were anticipated to exhibit a PDI close to 2 in line with previous studies [59]. Notably, all four samples exhibit a relatively narrow PDI, a characteristic often advantageous for industrial applications. A significant variance in molecular fraction within a polymer can influence its thermal properties, for example, [60]. The *M_n_* of PHBV is notably higher than the others, suggesting that PHBV may exhibit a higher melting temperature compared to PBAT, PBS, and PLA [61].

### 3.2. Thermal Properties

**Figure 4:**
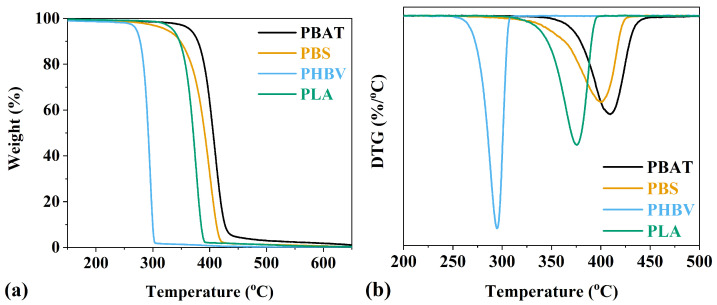
PBAT, PBS, PHBV, and PLA (**a**) TGA plots and (**b**) the maximum rate of degradation as shown by derivative thermogram (DTG).

The thermal properties of all polymers were investigated using DSC and TGA techniques. TGA analyzes weight changes with increasing temperature, providing insights into the thermal degradation behavior of each polymer sample. Figure 4 presents the thermal decomposition profile (a) and the derivative thermogram curve (b) for all four polymers. Table 2 details the thermal degradation temperatures (T_5_, T_10_, and T_20_ indicating 5%, 10%, and 20% weight loss, respectively). Notably, PBAT demonstrates greater stability compared to the others, with PHBV exhibiting the least thermal stability among the four polymers. This trend may be attributed to differences in the number-averaged molecular weight (*M_n_*) of the polymers, where PBAT has a lower *M_n_*, resulting in reduced chain mobility and fewer vulnerable end groups, thus enhancing its thermal stability. Conversely, PHBV, with higher *M_n_*, exhibits increased susceptibility to thermal degradation. The DTG analysis in Figure 4b enhances our understanding of the thermal decomposition kinetics, contributing to a comprehensive assessment of the thermal behavior of each polymer sample. PBAT exhibits a degradation peak at 410 °C, confirming its highest thermal stability among all the four polymers tested. In comparison, PBS and PLA show slightly lower maximum degradation rates at 400 °C and 375 °C, respectively. Notably, PHBV displays the lowest peak degradation temperature at 295 °C. This detailed thermal characterization provides valuable insights into the temperature ranges at which these polymers undergo decomposition, aiding in their application-specific considerations.

**Figure 5:**
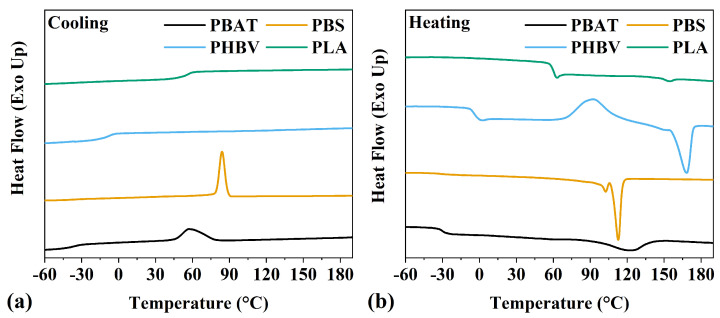
PBAT, PBS, PHBV, and PLA DSC thermogram results displaying (**a**) first cooling and (**b**) second heating.

Differential scanning calorimetry (DSC) is used to assess the thermal behavior of the polymer during heating and cooling cycles. The chemical structure of each polymer has a significant impact on its thermal behavior. Generally, polymers with a lower molecular weight exhibit enhanced thermal stability, as previously discussed. Additionally, molecular weight may also correlate with phase transitions, as fewer repeating units per chain can affect the ability of each polymer to undergo transitions such as melting or glass transition. However, the relationship between molecular weight and polymer phase transitions is complex and can be influenced by various factors, such as polymer composition, branching, and crystallinity. Figure 5 shows two cycles: the first cooling cycle, Figure 5a, and the second heating cycle, Figure 5b. As we can see from Figure 5 and the values reproduced in Table 2, PBAT, PBS, PHBV, and PLA samples have a melting temperature at 123 °C, 113 °C, 170 °C, and 154 °C, respectively. Noticeably, PBS has a double peak melting point (*T_m_*) in the second heating cycle, possibly due to the melt re-crystallization of the polymer [18]. PBS DSC curves display a smaller melting peak at 104 °C, and the second melting peak here is reported at 113 °C. Other researchers have reported this double melting peak with PBS as being due to fewer perfect crystals melting at lower temperatures, while more perfect crystals tend to melt at higher temperatures [62,63]. The glass transition temperature (*T_g_*) of PBAT, PBS, and PHBV samples follows a similar order as the melting temperature, with values of −30, −31, −4, and 54 °C, respectively.

Notably, PHBV and PLA display no exothermic peak corresponding to the crystallization (*T_c_*) at the cooling rate of 10 °C·min^−1^, indicating that both polymers, in this case, are primarily amorphous when cooling down from 200 °C. Similar to other studies [64], the heating cycle of PHBV and PLA displays a broad exothermic peak corresponding to the cold crystallization, with a peak at 86 °C for PHBV and 112 °C for PLA. This crystallization under heating occurs when the polymer is relatively quickly cooled into a disordered state, not having time to crystallize under cooling. Once the polymer is heated, the chains gain enough mobility to arrange themselves into crystallites [65].

The thermal and chemical properties of each polymer are correlated and confirmed by the literature. Polymers with a higher molecular mass tend to have lower T_5_ and higher *T_g_* [59,66]. The thermal results are essential to define the process and working temperature of the porous material. Below *T_g_*, because of the lack of mobility, polymers tend to be more brittle, and above *T_m_*, the polymers are more viscous than elastic. Therefore, the working temperature of a polymer is recommended to be between *T_g_* and *T_m_*. About processability, it is suggested to process the polymer above *T_m_* and below the T_5_ degradation temperature. Above T_5_, the polymer already loses 5% in mass and possibly does not comply with the properties of the pristine material.

### 3.3. Mechanical Properties

**Figure 6:**
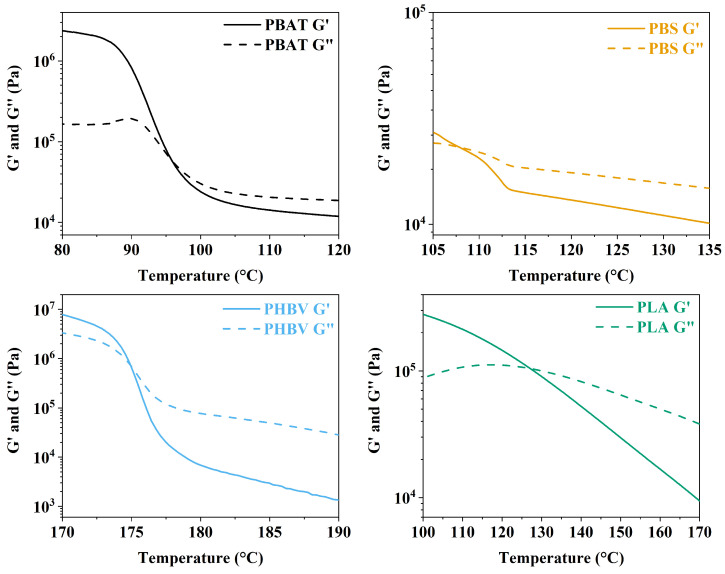
Modulus of PBAT, PBS, PHBV, and PLA samples as a function of temperature, measured at 1.0 rad·s^−1^ and 1.0% strain.

**Figure 7:**
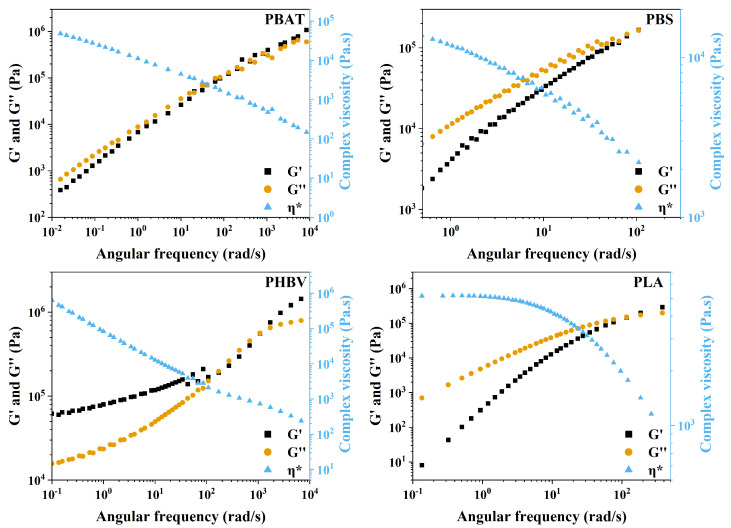
Modulus of PBAT, PBS, PHBV, and PLA samples as a function of angular frequency, measured at their respective melting temperatures (*T_m_*) and 1.0% strain.

The melt rheology of polymers helps us to understand the melting behavior of the polymer when forces are applied, deforming the material [67]. Therefore, it is common to correlate results from melt polymer rheological measurements to process or application parameters such as temperature, shear, and time. In addition, melt rheological measurements are often associated with molecular theories of polymer melt, including molecular weight, molecular distribution, and polymer chain behavior [68,69]. Rheological properties of PBAT, PBS, PHBV, and PLA were investigated and are shown in Figure 6 and Figure 7. Before the frequency and temperature sweep, amplitude sweep tests were performed in all four samples at different temperatures to identify each linear viscoelastic region (LVE) of the polymer. The LVE indicates the amplitude range where a frequency or temperature sweep can be performed with minimum influence over the entanglement or configuration of the polymer [70]. Figure 6 displays the modulus of the samples over temperature changes.

The intersection of the storage modulus (G′) and loss modulus (G″) typically aligns with the *T_m_* measured by DSC. As we move beyond this intersection toward higher temperatures, the samples undergo a transition toward increased viscosity and reduced elasticity. This delineates the processing region, which is especially crucial for the production of porous materials using supercritical fluid. However, notably, high viscosity during this phase may pose a risk to the structural integrity of the polymer. Notably, for PBAT, PBS, and PLA, the temperature of the G′ and G″ crossing is lower than their respective *T_m_* measured by DSC. In contrast, PHBV displays a delayed transition in rheological analysis compared to the DSC results. The discrepancy in transition temperature may arise from differences in sensitivity and measurement principles between rheological analysis and DSC. Therefore, the later transition observed in rheological analysis for PHBV implies additional complexities in its molecular structure or intermolecular interactions that affect mechanical behavior but are not fully captured by DSC. This emphasizes the need for a diversified approach to characterization, incorporating both thermal and mechanical analyses, to comprehensively understand the processing and application characteristics of polymers. In practice, all samples exhibit a consistent pattern, with the divergence in PHBV values correlating to the proximity of crystallization and melt peaks observed in DSC analysis. This aligns with the thermal characterization, indicating fewer perfect crystals in PHBV. The observed variations in rheological behavior thus find a correlation with the structural characteristics identified through thermal analysis, providing a cohesive understanding of the mechanical properties of the polymer. In order to produce a porous material from thermoplastics, the polymer needs to be in the region where it is more viscous than elastic so that the cells can expand and form the porous structure. The magnitude of the storage modulus of PBAT, PBS, and PLA samples after the crossing point toward higher temperatures dropped from 10^5^ to 10^4^; meanwhile, the magnitude of the PHBV modulus dropped from 10^6^ to 10^3^, showing a significant change in viscoelasticity over the temperature increase. Therefore, it is evident that in order to make a porous material with PHBV, it is necessary to have a process temperature closer to the *T_m_* of PHBV since it has a smaller processing window. Otherwise, the polymer can become more liquid-like, and it might not be able to hold its structure during or after the expansion, or may be too solid-like and not expand. PBAT, PBS, and PLA display a more stable modulus over temperature, meaning that they could have a foaming process at a temperature higher than *T_m_*.

Oscillatory measurements in Figure 7 characterize the viscoelastic behavior of each polymer at each respective *T_m_* measured by rheology. In this case, we use the time–temperature superposition (TTS) technique to assess each behavior of the polymers under a wider range of frequencies. TTS is a method for assessing the linear rheological properties of materials across a broad time or frequency range. This technique relies on the assumption that the underlying friction coefficient for all relevant relaxation processes, encompassing segmental and chain relaxation, remains constant. TTS is a valuable tool in rheological studies, enabling the extension of experimental data to conditions that may be challenging to achieve in the laboratory. The dependence of rheological properties on temperature is a crucial aspect, particularly when evaluating the actual serviceability of materials and investigating a larger time region at a specific temperature. TTS is not only instrumental in assessing linear rheological properties but also serves to evaluate polymer branching, homogeneity, miscibility, and phase separation in polymer blends. The validity of TTS is established when an exact superposition of shapes of adjacent rheological curves is achieved through horizontal and/or vertical shifts.

The data displayed in Figure 7 illustrate the frequency-dependent evolution of the G′ and G″ modulus, showcasing distinctive patterns for the studied polymers. Notably, PBAT, PBS, and PLA exhibit similar behavior, with their G′ and G″ crossover point occurring at nearly the same frequency. Before this intersection, G″ dominates over G′, indicative of a more viscous response, while, after the crossing, the modulus inverts, signaling a transition from terminal relaxation to a pseudo-rubbery plateau zone. This plateau suggests the presence of molecular interactions simulating a rubbery network over a specific frequency range. The observed behavior is crucial for understanding the material response during the expansion process for porous material production. The transition from viscous to elastic behavior at higher frequencies is influential, as it relates to the ability of the polymer to undergo fast and extensive deformation during the foaming process. In contrast, PHBV displays a distinct profile under different frequencies, particularly at its melting temperature. As shown in Figure 7, G′ starts higher than G", indicating a more elastic nature. However, as the frequency increases, there is a brief crossover where G″ surpasses G′, only to rapidly return to a more elastic behavior, where G″ becomes smaller than G′. This rapid transition can be identified as a transition zone. Unlike the other polymers, PHBV moves directly from the pseudo-rubbery plateau zone through the transition zone to the glass transition zone at *T_m_*. This behavior implies that, at its melting temperature, PHBV maintains a predominantly elastic response, making it less prone to fluidity or stretching required for pore formation during the foaming process. The abrupt shift from elastic to viscous behavior observed in Figure 6 after the melting point further emphasizes the challenges associated with foaming PHBV. The rheological analysis provides insights into the potential outcomes of the porous structure. However, assessing melt elasticity through small oscillatory shear strain may not be optimal, as it does not fully capture the high strain response observed during porous formation. Therefore, further investigation and foaming of each sample remain essential to comprehensively understand their behavior.

Figure 7 provides insight into the complex viscosity (η*) behavior of the samples at their respective *T_m_* across varying frequencies. A consistent trend is observed across all polymers, wherein the η* decreases with increasing frequency, a phenomenon known as shear thinning. This reduction in η* is attributed to the disentanglement of polymer chains occurring at higher shear rates. The relationship between η* and polymer chain entanglement is direct, with both parameters increasing as frequencies rise. Specifically, at the *T_m_* temperature, PBAT and PBS exhibit a similar η* profile from low to high frequencies. In contrast, PHBV and PLA display distinct shapes and magnitudes. PHBV demonstrates an almost linear drop from low frequencies, accompanied by higher η* at low frequencies compared to the other polymers. On the other hand, PLA exhibits a more logarithmic profile, with the lowest η* observed at lower frequencies compared to the other polymer tests. However, all four polymers exhibit a converging trend, displaying very similar magnitudes of η* at high frequencies.

### 3.4. Porous Materials

Porous materials were manufactured utilizing carbon dioxide for PBAT, PBS, PHBV, and PLA samples. Each sample underwent processing at its respective *T_m_*, under a pressure of 15 MPa, for 30 min, with depressurization lasting 2 to 4 s. The processing melt temperature was based in the reported data by the DSC and rheology results. Consequently, temperature emerged as a pivotal factor influencing polymer expansion. It is crucial to highlight that this study does not search for the mechanical or morphological properties of the porous material processed at different temperatures. Nevertheless, the chosen method holds significance in its utilization of CO_2_, aligning with sustainable practices. It is important to acknowledge that variations may occur at different temperatures and pressures. Under the specific conditions applied in this study, maintaining a pressure of 15 MPa, CO_2_ behaves as a supercritical fluid. This state of matter exhibits characteristics of both a liquid and a gas while sustaining a viscosity like that of a gas. This behavior of CO_2_ in its supercritical state enhances the plasticizing effect in thermoplastics during porous formation [71,72]. Recognizing these distinctive properties is essential for understanding and optimizing the porous material fabrication process, contributing to both the sustainability and efficiency of the chosen method. Supercritical CO_2_ is recognized for enhancing the plasticizing effect in thermoplastics during porous formation [71,72]. Given the uniform use of each respective *T_m_* of the polymer and a consistent pressure of 15 MPa, it is assumed that a similar plasticizing effect is employed across all four samples. This assumption further underscores the standardized conditions employed in this study, contributing to the reliability and comparability of the results.

**Figure 8:**
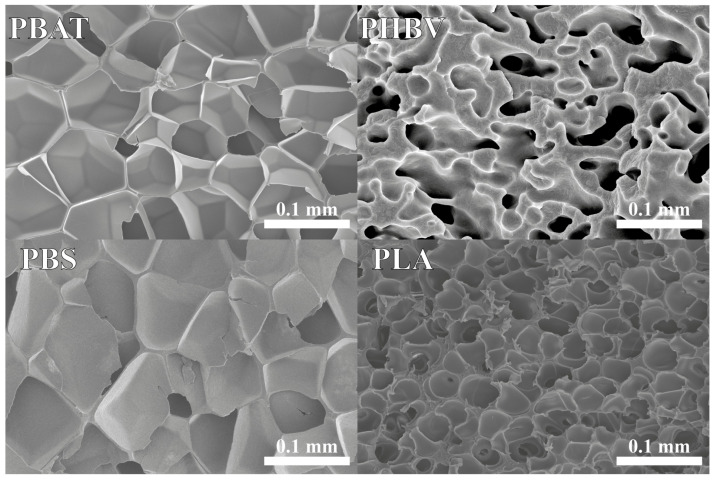
Representative SEM imaging of PBAT, PBS, PHBV, and PLA porous material foamed under equivalent processing conditions, resulting in different foam morphologies.

The morphological structures of PBAT, PBS, PHBV, and PLA samples are depicted in Figure 8. Notably, distinct characteristics are observed among the different polymers. PBAT and PBS predominantly feature closed, thin-walled porous structures, consistent with findings reported by previous studies [22,73]. Conversely, PHBV exhibits interconnected open pores with thicker walls, as demonstrated in other studies that utilized alternative approaches to achieve controlled foam structures [74]. PLA reveals small, closed pores with a combination of both thin and thick walls, which aligns with observations reported in other studies on PLA foams [75]. These variations in morphological features highlight the material-specific responses during the fabrication process using carbon dioxide as a foaming agent. The observed variations in pore size, connectivity, and wall thickness contribute to a nuanced understanding of how each polymer interacts with supercritical CO_2_ during the expansion process, ultimately influencing the resulting porous structure. In the case of PHBV, rheological results suggest challenges in stretching and pore formation, compelling CO_2_ to generate gaps between chains without forming well-defined channels and escaping the matrix. The observed limitations in the rheological response of the material, as discussed in the frequency sweep analysis, contribute to a more interconnected and more constrained porous structure during the expansion process. While anticipations might suggest a more solid-like behavior for PHBV at *T_m_*, the plasticizing effect of CO_2_ significantly influences the material, lowering its transition temperature. The combination of this plasticizing effect and the abrupt drop in storage modulus observed in the temperature sweep further supports the notion that PHBV struggles to achieve full stretching and the formation of interconnected pores. Consequently, the morphological structure of PHBV porous material differs from that of PBAT, PBS, and PLA.

In spite of PBAT and PBS sharing similar melt mechanical properties, subtle differences arise in their porous structures. PBAT exhibits thinner walls and a more hexagonal shape in its pores, whereas PBS tends to have thicker walls and a rounder pore shape compared to PBAT. PLA, on the other hand, showcases more distinctive features, with smaller, rounder pores and even thicker walls than PBAT and PBS. These variations in porous morphology among PBAT, PBS, and PLA stem from various factors previously described. Chemical structure differences, identified through FTIR and NMR analyses, highlight the impact of specific bonds and functional groups. Molecular weight, as revealed by GPC analyses, also plays a significant role in affecting viscosity, melt flow behavior, and phase separation during processing. Additionally, rheological measurements underscore the importance and interconnection of molecular weight in shaping polymer behavior. Higher entanglement densities result in greater resistance to deformation, further impacting the porous structure. In summary, the porous morphology of these polymers reflects a complex interplay of chemical structure, molecular weight, thermomechanical behavior, and polymer entanglements as discussed in previous sections.

**Figure 9:**
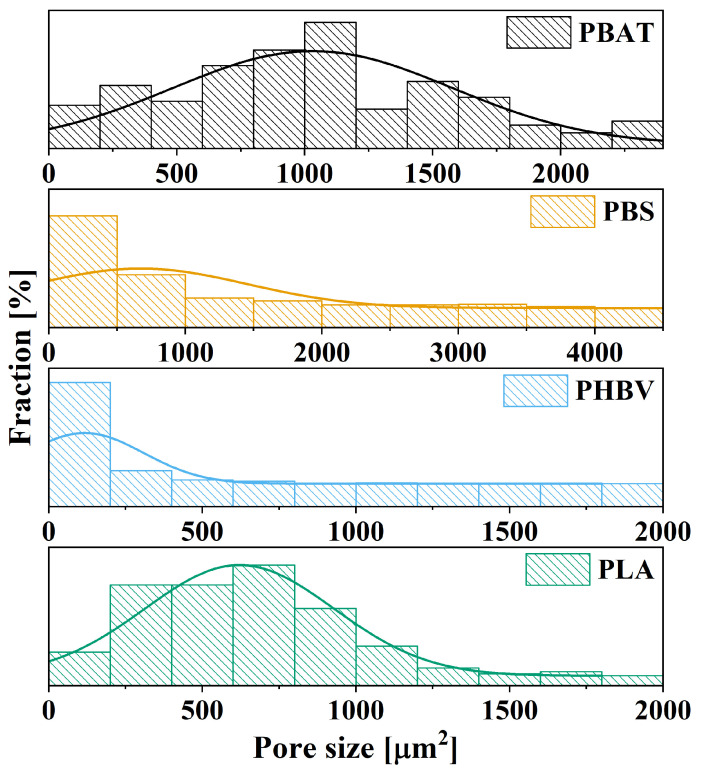
Cell size distributions of PBAT, PBS, PHBV, and PLA foamed at their respective *T_m_* and 15 MPa.

The size and distribution of pores play a crucial role in determining the potential applications of materials, impacting not only their morphology but also their mechanical and thermal properties. Homogeneous nanoporous structures, for instance, find applications in fields such as tissue engineering, membranes, and catalytic reactions [76,77]. Complex porous structures, encompassing both micro and nanostructures, offer versatility by combining large pores for reduced bulk density with smaller pores that enhance mechanical and thermal properties. This makes them well suited for applications such as thermal insulation and electromagnetic shielding [78,79]. In Figure 9, the histogram illustrates the pore size distribution for PBAT, PBS, PHBV, and PLA. PBS exhibits an average porous area of 673 μm^2^ with a standard deviation of 780 μm^2^, representing the least homogeneous distribution among the tested samples. The wide range of porous areas, spanning from 100 μm^2^ to over 4000 μm^2^, may be attributed to melting strength or, as suggested by double-peak DSC results, potential re-crystallization in this temperature range. The double melting peaks in PBS indicate a complex thermal behavior that may result in dissimilarities in porous morphology, influenced by variations in molecular chain alignment at *T_m_*. PHBV, with an average cell area of 116.6 μm^2^ and a standard deviation of 192 μm^2^, displays the least dispersion in cell size. This can be attributed to its restricted stress endurance capacity, resulting in channels or interconnected pores with similar areas. PBAT exhibits a relatively homogeneous distribution, with an average porous area of 1030.86 μm^2^ and a standard deviation of 544 μm^2^. Similarly, PLA demonstrates a uniform distribution, comparable to PBAT, with an average area of 620 μm^2^ and a standard deviation of 307 μm^2^. Notably, PBS and PHBV exhibit larger standard deviations compared to their respective means, while PBAT and PLA display smaller standard deviations relative to their means. These variations highlight the diverse and unique pore size distributions of each polymer, providing insights into their potential applications and material behaviors.

## 4. Conclusions

In this study, we comprehensively characterized thermoplastics PBAT, PBS, PHBV, and PLA, aiming to elucidate the relationships between their properties and the formation of porous materials. While our findings offer insights into the behavior of these polymers under foaming conditions, establishing direct correlations between polymer properties and foam behavior remains challenging. Thermal analysis provided critical data on melting temperatures for processing with scCO_2_, while rheological measurements aided in understanding melt behavior during foaming. Foaming experiments validated hypotheses derived from chemical, thermal, and mechanical tests, displaying distinct morphological outcomes for each polymer. PBAT, PBS, and PLA exhibited closed-pore structures, while PHBV formed interconnected channels. Quantitative measurements of porous area supported these findings, with PBAT foam demonstrating an average porous area of 1030.86 μm^2^, PBS foam at 673 μm^2^, PLA foam at 620 μm^2^, and PHBV foam featuring interconnected channels with an average area of 116.6 μm^2^. However, correlating these findings directly with polymer properties remains complex. The characterized thermoplastics exhibit diverse properties that position them as versatile materials in porous structures, with applications ranging from lightweight structures to environmentally friendly solutions. The use of scCO_2_ as a foaming agent and plasticizer offers a green and sustainable approach, facilitating the design of diverse porous structures. However, limitations persist in predicting polymer behavior under foaming conditions using physical blowing agents. In conclusion, while our study provides insights and contributions to the field, coupled with the innovative scCO_2_ process, challenges remain in predicting polymer behavior under foaming conditions. Further research is needed to address these limitations and enhance our understanding of foam formation in thermoplastics.

## Figures and Tables

**Table 1:** Number-averaged molecular weight (*M_n_*), weight-averaged molecular weight (*M_w_*) given in kDa, and the polydispersity index (PDI) of PBAT, PBS, and PHBV.

Polymer	*M_n_* (kDa)	*M_w_* (kDa)	PDI
PBAT	52	132	2.5
PBS	100	233	2.3
PHBV	207	440	2.1
PLA	105	189	1.8

**Table 2:** Summary of thermal properties of PBAT, PBS, PHBV, and PLA measured using DSC and TGA techniques.

Sample	*T_g_* (°C)	*T_c_* (°C)	*T_m_* (°C)	T_5_ (°C)	T_10_ (°C)	T_20_ (°C)
PBAT	−30	74	123	367	380	391
PBS	−31	84	113	323	347	367
PHBV	−4	86 *	170	225	275	283
PLA	54	112 *	154	335	347	357

* *T_cc_* (cold crystallization temperature).

## Data Availability

The data presented in this study are available in this article.
